# Investigating REPAIRv2 as a Tool to Edit *CFTR* mRNA with Premature Stop Codons

**DOI:** 10.3390/ijms21134781

**Published:** 2020-07-06

**Authors:** Raffaella Melfi, Patrizia Cancemi, Roberta Chiavetta, Viviana Barra, Laura Lentini, Aldo Di Leonardo

**Affiliations:** Department of Biological, Chemical and Pharmaceutical Sciences and Technologies (STEBICEF), University of Palermo, 90128 Palermo, Italy; patrizia.cancemi@unipa.it (P.C.); roberta.chiavetta@unipa.it (R.C.); viviana.barra@unipa.it (V.B.); laura.lentini@unipa.it (L.L.)

**Keywords:** cystic fibrosis, premature termination codons (PTCs), RNA editing, CRISPR/dCas13b

## Abstract

Cystic fibrosis (CF) is caused by mutations in the gene encoding the transmembrane conductance regulator (CFTR) protein. Some CF patients are compound heterozygous or homozygous for nonsense mutations in the *CFTR* gene. This implies the presence in the transcript of premature termination codons (PTCs) responsible for a truncated CFTR protein and a more severe form of the disease. Aminoglycoside and PTC124 derivatives have been used for the read-through of PTCs to restore the full-length CFTR protein. However, in a precision medicine framework, the CRISPR/dCas13b-based molecular tool *“REPAIRv2” (RNA Editing for Programmable A to I Replacement, version 2*) could be a good alternative to restore the full-length CFTR protein. This RNA editing approach is based on the targeting of the deaminase domain of the hADAR2 enzyme fused to the dCas13b protein to a specific adenosine to be edited to inosine in the mutant mRNA. Targeting specificity is allowed by a guide RNA (*g*RNA) complementarily to the target region and recognized by the dCas13b protein. Here, we used the REPAIRv2 platform to edit the UGA PTC to UGG in different cell types, namely IB3-1 cells, HeLa, and FRT cells engineered to express H2BGFP*^opal^* and CFTR*^W1282X^*, respectively.

## 1. Introduction

Cystic fibrosis (CF) is caused by mutations in the gene encoding the cystic fibrosis transmembrane conductance regulator (CFTR) protein, an ATP-gated anion channel, which plays a major role in regulating both secretion and absorption in many epithelial tissues [1]. Nonsense mutations in the *CFTR* gene generate premature termination codons (PTCs), leading to premature translation termination and causing the synthesis of a truncated non-functional CFTR protein.

Approximately 8% of CF patients worldwide and 13% in Europe were reported to be compound heterozygous or homozygous for a nonsense mutation and the percentage of these patients was remarkably high in Israel (*Ashkenazi* 45.5%), Italy (32.4%), and Slovenia (27.3%) [2].

These patients could take advantage of a drug treatment promoting the translation read-through of the PTC to restore the CFTR full-length protein, at least to some extent. In the past, aminoglycoside antibiotics (e.g., G418) were used for the read-through approach since they affect the proofreading function of the ribosome [3]. However, prolonged treatments with aminoglycosides cause severe side effects [4], thus their clinical use has been limited. New aminoglycosides like NB30 and NB54, as well as the unrelated chemical compound PTC124, have been reported to promote the read-through of PTCs, likely leading to the insertion of a near-cognate amino acid at the PTC site in the CFTR protein [5,6]. However, PTC124 (also known as Ataluren) has been discontinued as a potential drug to treat FC after phase 3 trial and failure to restore CFTR in organoids [7]. Recently, some small molecules, PTC124 derivatives that do not show the toxicity of aminoglycosides, have been suggested as a potential treatment for genetic disorders caused by nonsense mutations [8,9,10,11,12,13].

Although the development of drugs aimed to overcome nonsense mutations appears promising [11,12], it cannot be ruled out the possibility that they introduce a non-cognate amino acid, which impairs full-length protein function. Thus, novel approaches based on site-directed RNA editing (SDRE) to restore the full-length functional protein are worth to be investigated.

Different SDRE promising platforms have been reported in recent literature [14,15,16,17]. Some are based on recruitment of native adenosine deaminases acting on RNA (ADARs) by antisense oligonucleotides (ASOs) [18,19,20], others on the recruitment of exogenous recombinant ADARs via the fusion to an RNA guide recognizing domain. The RNA guide hybridizes to the target region where the deaminases are thus conveyed [21,22].

The Rosenthal lab [23] first reported the use of SDRE to correct the *CFTR* mRNA with the W496X nonsense mutation injected into Xenopus oocytes.

In the era of personalized medicine, the CRISPR (classes of regularly interspaced short palindromic repeats) and CAS (CRISPR-associated) nuclease systems are considered valuable tools for novel therapeutics’ approaches [24]. Recently, the Feng Zhang lab pointed out the possibility of editing mRNA at the level of specific stop mutations before it undergoes translation. This has been made possible by exploiting the deaminase domain (DD) of the human ADAR2 [25]. Human ADAR2 changes adenosine (A) into inosine (I) by hydrolytic deamination. By this novel technology, the “*RNA Editing for Programmable A to I Replacement, version 2*” (REPAIRv2), editing is achieved by directing the ADAR2_DD_ (E488Q/T375G) to mutant mRNAs by the catalytically inactive CRISPR-associated RNA-guided RNase Cas13b (dCas13b) and a 25–80-nucleotides-long specific guide RNA (*g*RNA). Editing the adenosine of the PTC (UGA) to inosine would correct the nonsense mutation since the ribosomal machinery reads inosine as guanosine. Thus, for mutations involving a G>A transition, deamination can restore a sense codon or even the WT coding sequence in place of a premature stop codon.

A concern of ADAR’s overexpression is that the system could be associated to a higher risk of off-target activity, which is still a major issue in SDRE [26]. However, the REPAIRv2 platform was able to reduce the detectable off-target edits from about 8500 (λN-BoxB) to only 20 in the whole transcriptome [15], providing higher specificity than previously described RNA editing platforms.

It is important to remark that, in contrast to DNA editing whose off-target effects are unpredictable and potentially hazardous, RNA editing is transient, can be more easily reversed, allowing a better control over editing outcomes, and it does not require the presence, at the targeting sites, of a protospacer adjacent motif (PAM) [27].

Here, we evaluated the REPAIRv2 platform efficacy in editing the UGA premature termination codon in the H2BGFP*^opal^* reporter gene [28] and in the *CFTR^W1282X^* cDNA. We first targeted an UGA PTC that was previously introduced in the transcript coding for the H2B histone tagged with the green fluorescent protein (H2BGFP) expressed in engineered HeLa cells (HeLa-H2BGFP*^opal^*) [10], where it decorates the chromosomes in green [29]. We then introduced the W1282X nonsense mutation in the *CFTR* cDNA and then engineered Fischer Rat Thyroid cells (FRT-CFTR*^W1282X^*) as a model to edit the *CFTR* transcript and restore the full-length protein. Furthermore, we edited the naturally occurring nonsense mutation in human IB3-1 airway epithelial cells (compound heterozygous F508del/W1282X) that do not express any detectable amounts of endogenous CFTR protein [30].

## 2. Results

### 2.1. ‘Spacer’ Design and Cloning into the Vector pC0043-PspCas13b

**Figure 1:**
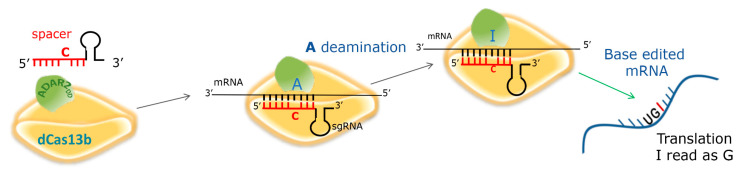
Schematic representation of mRNA editing by REPAIRv2; sgRNA: Specific *guide* RNA. The C:A mismatch at the target position and the outcome of editing, A to I replacement in the mRNA, are shown.

The catalytic domain of the hADAR2 deaminase (ADAR2_DD_) is fused to the dCas13b protein necessary for the recognition of the specific target. To address the dCAS13b/ADAR2_DD_ fusion protein to the target mRNA, cells must also express a *guide* RNA (*g*RNA). The *g*RNA is composed of a *spacer*, a specific sequence complementary to the mRNA region surrounding the adenine to be deaminated, which is recognized by a C:A mismatch, followed by a *scaffold*, a sequence that folds to create the recognition hairpin for dCas13b (Figure 1).

*Spacers* were obtained by annealing two synthetic complementary single-strand oligonucleotides 30 and 50 bases long, considering that *spacers*’ length could affect editing [25]. The C base at the target position in the forward oligo (or antisense strand) was introduced at different distances from the 3′ end of the *spacer* and, thus, from the 5′ end of the scaffold, to generate the C:A mismatch in the hybrid gRNA/mRNA. This distance between the target adenine and the first nucleotide of the scaffold, indeed, seems to affect the recognition of the correct adenine to target and, consequently, the precision of the editing. Finally, in the 5′ position of the spacer we made sure to have a guanine, which is important to enhance the transcription from the U6 promoter, and to have different 5′ overhangs allowing directional cloning [25].

We first aimed at targeting the premature stop codon TGA that causes the mutation W190X in the H2BGFP protein [10]. Editing the mRNA by changing the UGA into UGI is supposed to restore the tryptophan at position 190 in the polypeptide chain, suppressing the nonsense mutation. Four sets of complementary oligonucleotides were designed to obtain specific spacers to target the adenine at position 570 of the H2B-GFPopal cDNA. Three spacers were 50 bases long and the distance of the mismatch position from the 3′ ends was of 32, 34, or 35 nucelotides (nts), respectively. The last spacer was 30 bases long and the distance of the mismatch was of 25 nts (Supplementary Figure S1A–D). The same approach was used to target the W1282X opal nonsense mutation (UGG>UGA, g3846a) in the *CFTR* mRNA. To this aim, we designed three different 50-nucleotides-long spacers, complementary to the region surrounding the W1282X mutation. In the gRNA/mRNA hybrid the distance of the C:A mismatch from 3′ end of the spacer was of 32, 34, and 35 nts (Supplementary Figure S2A–C).

The annealed complementary oligonucleotides were cloned into the BbsI sites of PspCas13b crispr RNA (crRNA) backbone vector (Addgene) upstream of the scaffold sequence, under the control of the U6 promoter (Figure S3). Positive clones were selected by colony PCR with different couples of primers for GFP *g*RNA clones and CFTR *g*RNA clones (Supplementary Figure S4A,B).

### 2.2. Evaluation by Fluorescence Microscopy of RNA Editing of the H2BGFP^opal^ Stop Mutation

**Figure 2:**
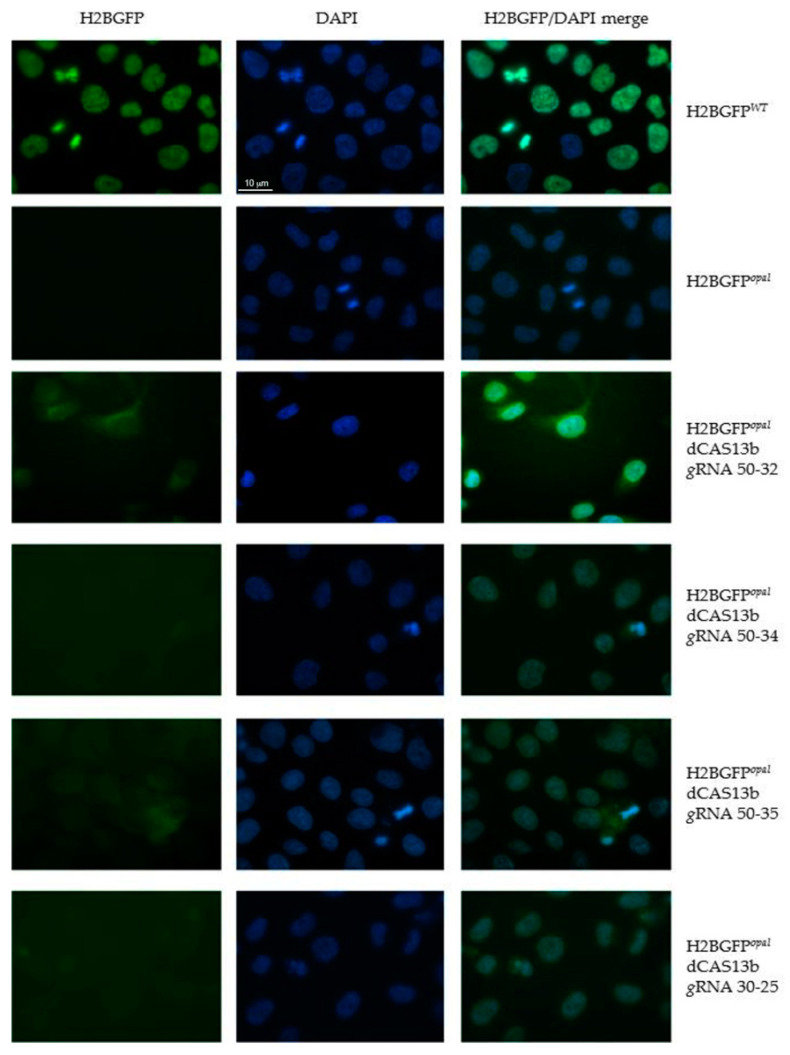
Fluorescence analysis to detect editing of the PTC (UGA) in HeLa cells expressing the H2BGFP*^opal^* gene. HeLa-H2BGFP*^opal^* cells were co-transfected with dCAS13b/ADAR2_DD_ and selected *g*RNAs coding plasmids (450 ng total DNA). Two days after transfection, cells were fixed with methanol for 5 min and the green fluorescence due to H2BGFP expression was monitored using the 63X objective on a ZEISS microscope equipped for epifluorescence. Nuclei were visualized with DAPI (blue). HeLa H2BGFP*^WT^* cells and untransfected H2BGFP*^opal^* cells were used as positive and negative control, respectively.

The HeLa-H2BGFP*^opal^* cells were transiently transfected with the dCas13b/ADAR2_DD_ plasmid and with the plasmid carrying specific GFP *guide* RNAs. To optimize the experimental procedure, we did several transfections varying the amount of the two plasmids, maintaining the 2:1 ratio (*g*RNA: dCAS13b/ADAR2_DD_). After 48 h from the transfection, cells were fixed and observed at the fluorescence microscope. Green fluorescence was observed both in the nuclei of HeLa H2BGFP*^WT^* cells, as expected, and in the transfected HeLa H2BGFP*^opal^* cells, although in these cells the fluorescence appeared less bright (Figure 2).

**Figure 3:**
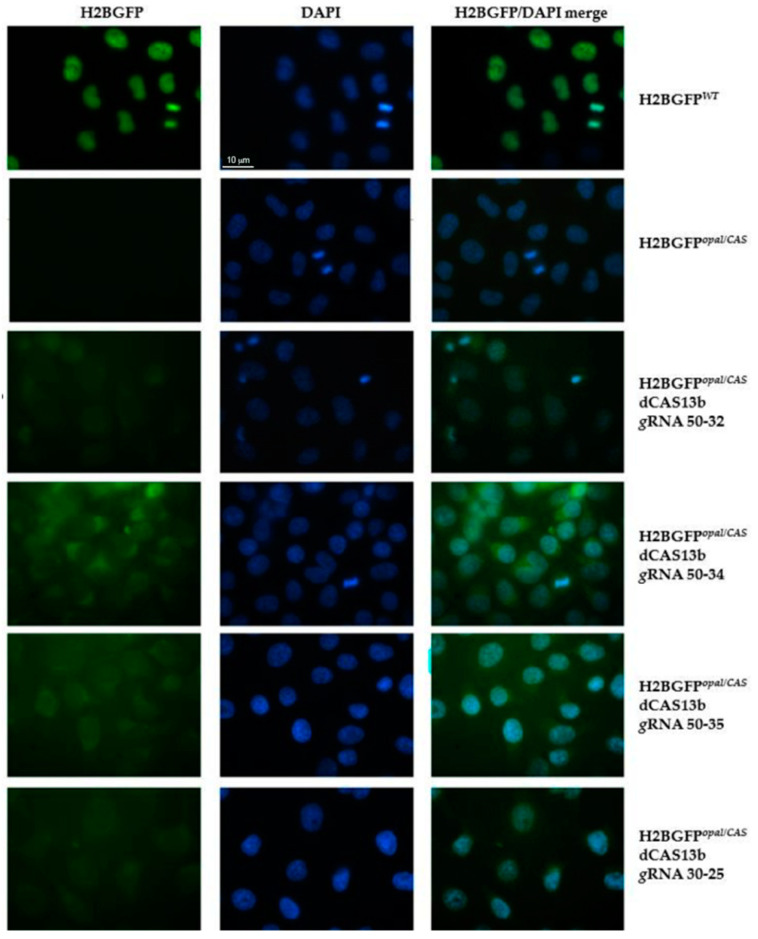
Fluorescence analysis after editing the PTC (UGA) in HeLa-H2BGFP*^opal^* cells stably expressing dCAS13b/ADAR2_DD_. HeLa cells stably expressing dCAS13b/ADAR2_DD_ were transfected with selected *g*RNAs coding plasmids (300 ng of total DNA). Two days after transfection, cells were fixed with methanol for 5 min and the H2BGFP (green) was detected using the 63x objective on a ZEISS microscope equipped for epifluorescence. Nuclei were stained with DAPI (blue). HeLa-H2BGFP*^WT^* cells and untransfected HeLa-H2BGFP*^opal^* cells, expressing dCAS13b/ADAR2_DD_, were used as positive and negative control, respectively.

The modest level of editing observed in these experiments could be explained by the low co-transfection efficiency of the REPAIRv2 system plasmids. Thus, in the attempt to increase the chance of editing the UGA premature termination codon by the REPAIRv2 system, we generated HeLa-H2BGFP*^opal^* cells stably expressing dCAS13b/ADAR2_DD_. After transfection with selected *g*RNAs coding plasmids, green fluorescence was brighter than in the cells transfected with both plasmids in transient. H2BGFP was detectable in HeLa-H2BGFP*^opal^* cells, in particular in those transfected with the GFP *g*RNAs 50–34 or 50–35 clones, though to a lesser extent than in HeLa-H2BGFP*^WT^* used as control. Lower GFP fluorescence was detected in HeLa-H2BGFP*^opal^* cells expressing the *g*RNAs 50–32 or 30–25 (Figure 3).

A difference in editing efficacy of different guides can be expected since, as stated above (Section 2.1), it is known that the distance of the target adenine from 3′ end of the spacer may affect the ability of the ADAR deaminase to contact it. Following our observations, for the experiments described in the next sections we used 50-nucleotides-long *g*RNAs with the mismatch distances from 3′ end of 34 and 32 nucleotides (*g*RNA 50–34 and a *g*RNA 50–32, respectively) that, at least in the case of H2BGFP*^opal^* mRNA, seemed to give better results.

**Figure 4:**
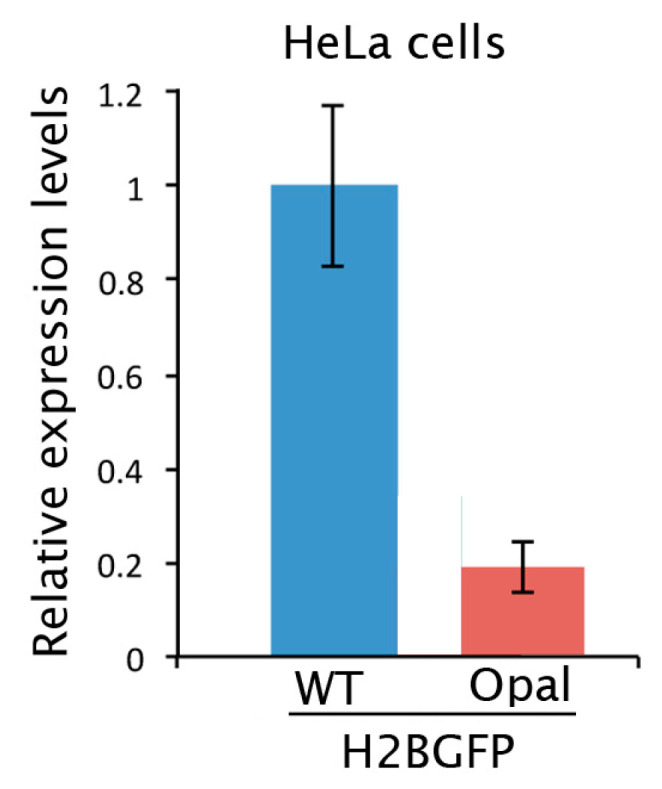
H2BGFP gene expression levels measured by RT-qPCR in HeLa-H2BGFP^WT^ and H2BGFP^opal^ cells. The histogram represents the mean ± SD of a quadruplicate.

Another factor that could affect the level of editing is the amount of the target mRNA to be edited in the cells. Indeed, in these cells we observed that mRNA with nonsense codon is less abundant than the WT (Figure 4). Although here H2BGFP*^opal^* is expressed from a cDNA and it is not subjected to splicing, and thus not controlled by nonsense-mediated decay (NMD) [31], its mRNA probably undergoes the mRNA no-go decay (NGD) surveillance pathway that is triggered when ribosomes stall on damaged mRNA or an mRNA harboring a PTC [32,33].

### 2.3. Editing of the CFTR Transcripts with UGA Nonsense Mutation in FRT-CFTR^W1282X^ Cells

**Figure 5:**
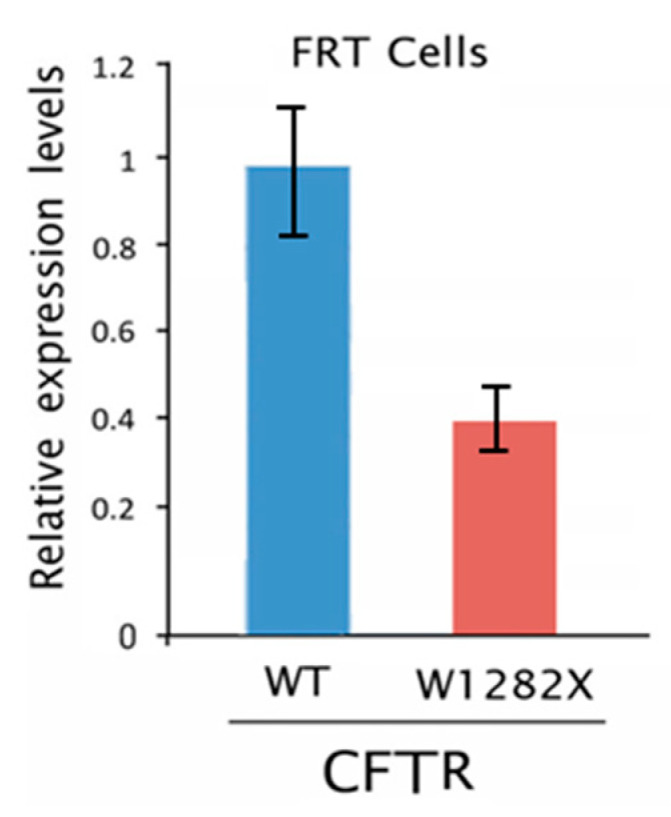
*CFTR* gene expression level evaluated by RT-qPCR in FRT-CFTR*^WT^* and FRT-CFTR*^W1282X^* stably transfected cells. The histogram represents the mean ± SD of a quadruplicate.

To evaluate the efficacy of the REPAIRv2 system on *CFTR* transcript with PTC, we used the engineered FRT epithelial cells, which do not express endogenous cyclic AMP (cAMP)-regulated chloride (Cl-) channels [34], stably transfected with a plasmid encoding a nonsense mutant *CFTR* transcript (*CFTR^W1282X^*). To this aim, in the full-length *CFTR* cDNA that is cloned in the pTracer plasmid (pTCF-WT) [34,35], we introduced a premature stop codon (TGG>TGA) in place of the Tryptophan 1282-coding codon (W1282) by site-directed mutagenesis. By colony PCR, we isolated a positive bacterial clone. Purified plasmid DNA was verified by sequencing (Figure S5A,B) and then stably transfected in FRT cells. The amount of the *CFTR^W1282X^* mutant transcript, representing the target for editing in FRT-CFTR*^W1282X^*cells, was assessed by RT-qPCR and it was less than 40% of the *CFTR^WT^* transcript (Figure 5). The reduction of this transcript harboring a PTC was probably caused by the NGD pathway, as suggested above. 

**Figure 6:**
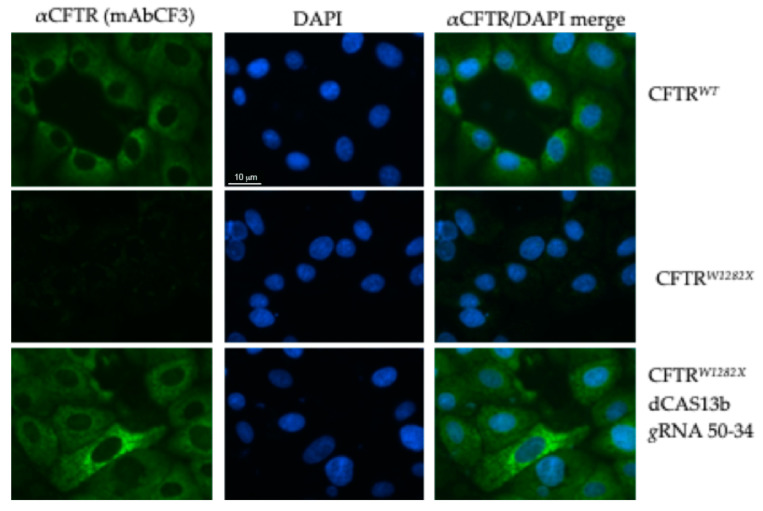
Immunofluorescence detection of CFTR protein in FRT-CFTR*^W1282X^* cells. Cells untransfected (negative control) and transfected with the plasmids encoding the indicated *g*RNA and Cas13b/ADAR2_DD_ (300 ng total DNA) are shown. FRT-CFTR*^WT^* cells were used as a positive control. Cells were fixed with methanol and the CFTR protein was revealed by the primary antibody mAbCF3, followed by a secondary antibody anti-mouse Alexa-488, (green, Abcam). Nuclei (blue) were DAPI stained. Images were taken at 100x magnification on a ZEISS microscope equipped for epifluorescence.

**Figure 7:**
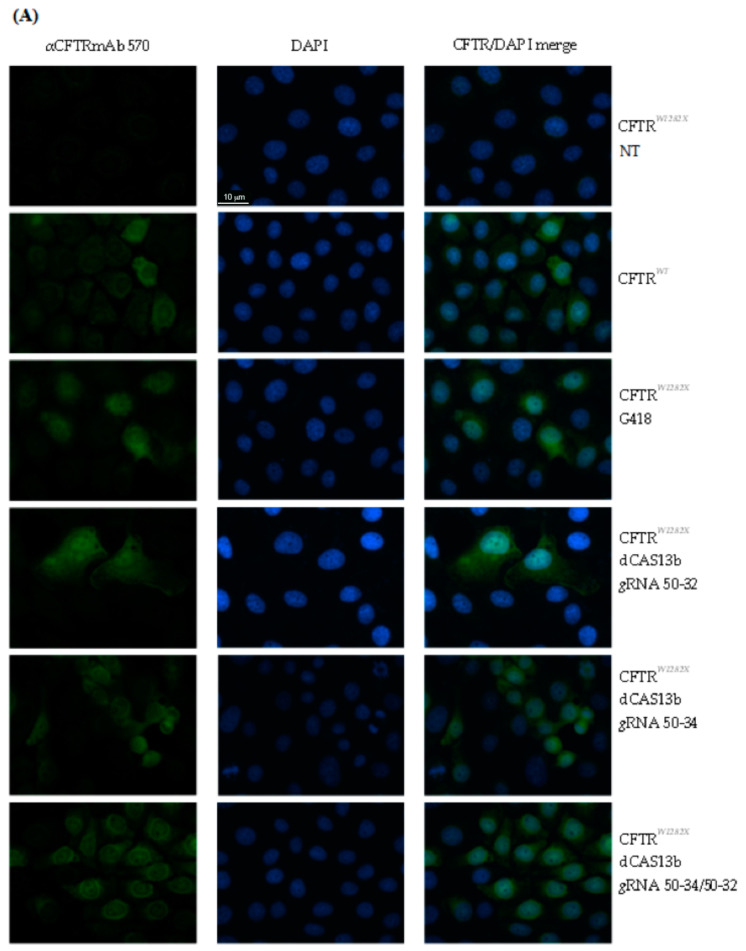

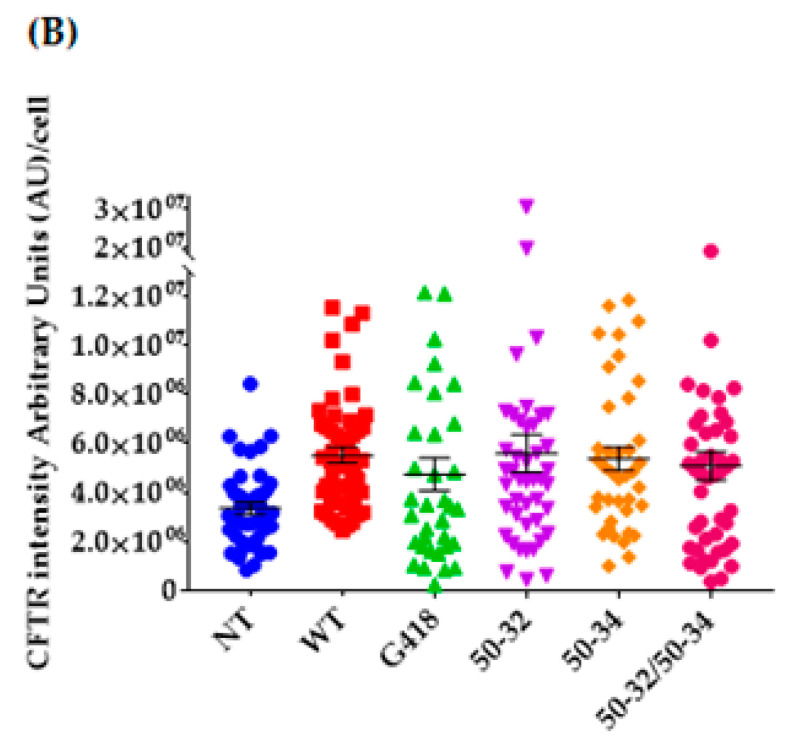
Immunofluorescence analysis of CFTR protein in triton permeabilized FRT-CFTR^W1282X^ cells and quantification of the immunofluorescence. FRT-CFTR*^W1282X^* cells were transfected with the plasmids encoding the indicated *g*RNAs and dCAS13b/ADAR2_DD_ (300 ng total DNA). Untransfected FRT-CFTR*^W1282X^* cells (NT) were used as negative control. FRT-CFTR*^W1282X^* cells treated with G418 and FRT-CFTR*^WT^* cells were used as a positive control. (**A**) The CFTR protein was revealed by the primary antibody mAb570, followed by a secondary antibody anti-mouse-FITC conjugated (green, Sigma). Nuclei (blue) were DAPI stained. Images were taken at 63x magnification on a ZEISS microscope equipped for epifluorescence. (**B**) The quantification is relative to the replicate representative of the results. The error bar represents the standard error of the mean (SEM). The quantification of CFTR signal was done manually by using Fiji software. The cell contour was drawn by using a cell membrane marker. The background was subtracted and the integrated signal intensity in the selected area (one cell) was measured.

To edit the mutant mRNA and rescue the full-length CFTR protein, FRT-CFTR*^W1282X^*cells were transfected with plasmids coding for the dCas13b/ADAR2_DD_ fusion protein and with selected CFTR *g*RNAs. To optimize the experimental procedure, we did several transfections, varying the amount of the two plasmids, always maintaining the 2:1 ratio (*g*RNA:dCAS13b). The CFTR protein was then detected using different antibodies: mAbCF3, targeting the extracellular loop of CFTR, and mAb570, targeting the intracellular R domain of the protein. With the aim to enhance the antibody entry into the cells and epitope binding, cells fixed with methanol were further permeabilized with triton treatment before the incubation with mAb570. However, as shown in Figures S6 and S7, no significant difference was observed when only methanol fixation was performed. We detected positive cells for the CFTR protein by immunofluorescence microscopy (Figure 6 and Figure 7A), suggesting that there the amount of the *CFTR^W1282X^* transcript was enough for the REPAIRv2 tool to work. First, cells were transfected with the dCAS13b/ADAR_DD_ vector and single CFTR *g*RNA 50–34 clone (Figure 6).

Next, in the attempt to maximize the editing, we simultaneously transfected the cells with both CFTR *g*RNAs 50–34 and 50–32, maintaining the 2:1 ratio (*g*RNA:dCAS13b/ADAR2_DD_). As shown in Figure 7A,B, the number of positive cells was appreciably higher if compared with the transfections with a single *g*RNA plasmid.

These results show that although the amount of target *CFTR*’s mutant mRNA is low, the REPAIRv2 system provided enough editing to allow the rescue of the CFTR protein, at least in some cells.

### 2.4. Evaluation of CFTR Rescue Following Editing CFTR^W1282X^ in IB3-1 Human Cells

Encouraged by the results obtained with FRT-CFTR^W1282X^ cells, we then evaluated if the REPAIRv2 system could be able to edit the endogenous *CFTR*^W1282X^ mutation present in IB3-1 airway epithelial cells (compound heterozygous F508del/W1282X) that do not express any detectable amounts of endogenous CFTR protein [30].

By using REPAIRv2 in IB3-1 airway epithelial cells, the recovery of CFTR protein will only be the result of the UGA>UGG conversion by RNA editing, since, to correct the F508del *CFTR* mRNA, it would be necessary to insert a triplet in the mutant transcript, a task that this technology is unable to do.

**Figure 8:**
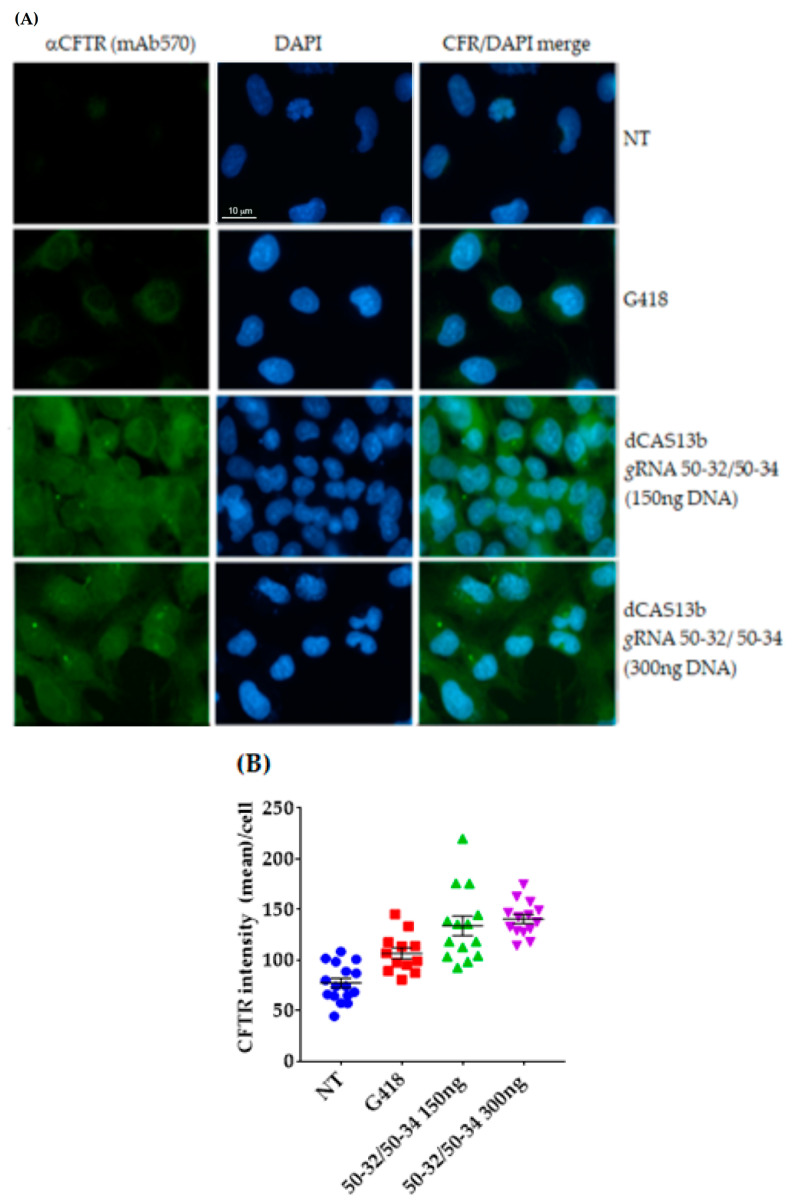
Immunofluorescence analysis to detect the CFTR protein in triton permeabilized IB3-1 cells and quantification of the immunofluorescence. IB3-1 cells were untreated (NT: Negative control), treated with G418 (positive control), or transfected with the plasmids encoding the 50-32/5034 *g*RNAs and dCAS13b/ADAR2_DD_. (**A**) CFTR protein was revealed by the specific primary antibody mAb570 and a secondary antibody (green, Alexa-488, Abcam). Nuclei (blue) were DAPI stained. Images were taken at 63x magnification on a ZEISS microscope equipped for epifluorescence. (**B**) The quantification is relative to the replicate representative of the results. The error bar represents the SEM. The quantification of CFTR signal was done manually by using Fiji software. The cell contour was drawn by using a cell membrane marker. The background was subtracted and the pixel mean in the selected area (one cell) was measured.

Taking advantage of our previous results, we performed transfections with two CFTR *g*RNAs’ clones, 50–32 and/or 50–34, at two different concentrations and we then detected the CFTR protein with the specific antibody mAb570 by immunofluorescence microscopy. Different from what we observed in the experiments using FRT-CFTR*^W1282X^,* the majority of IB3-1 cells were fluorescent, indicating the presence of the CFTR protein following editing with the two different *g*RNAs, irrespective of the total amount of transfected DNA (Figure 8A,B).

### 2.5. Visualization of Editing by cDNA Sequencing

**Figure 9:**
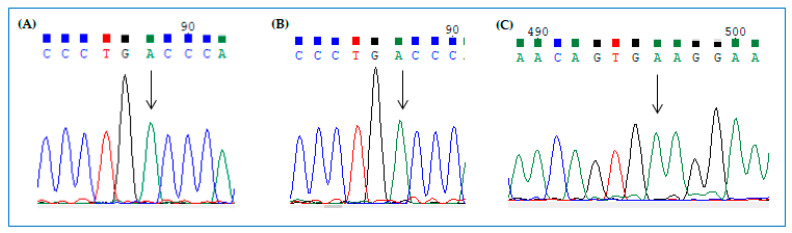
Electropherograms of sequenced H2BGFP*^opal^* and *CFTR^W1282X^* amplicons from cells expressing (**A**) GFP *g*RNA 50–34, (**B**) GFP *g*RNA 50–35, (**C**) CFTR *g*RNA 50–34. Mutant positions are indicated by the arrows.

Forty-eight hours after cell transfection with Cas13b/ADAR2_DD_ vector and *g*RNA clones, total RNA was extracted and retrotranscribed. The cDNAs were then amplified with primers surrounding the mutant position and sequenced (BMR genomics) to verify if any editing occurred. In the case of effective editing, we expected to see a double A/G peak at the mutant position in the electropherograms. Representative experiments with HeLa-H2BGFP*^opal^* cells and FTR-CFTR*^W1282X^* are shown here. To edit the UGA PTC, the two cell lines were transfected with dCas13b/ADAR2_DD_ vector and plasmids encoding GFP *g*RNA 50–34 or 50–35 and CFTR *g*RNA 50–34, respectively. Following RNA extraction, retrotranscription, and PCR amplification, by standard sequencing we were unable to detect a double A/G peak at target positions in the electropherograms of H2BGFP*^opal^* reporter gene (Figure 9A,B) and *CFTR^W1282X^* (Figure 9C).

**Figure 10:**
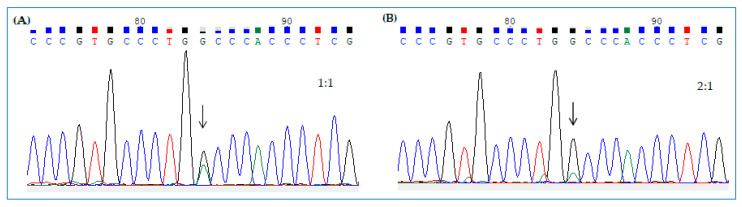
Electropherograms relative to H2BGFP WT/opal amplicons’ mix at 1:1 (**A**) and 2:1 ratio (**B**). The double peaks are indicated by the arrows.

These results suggest that the amount of edited transcript was below the threshold of sensitivity of the assay to detect a double A/G peak, which, instead, was visible when we sequenced a mix of H2PGFP WT and opal mutant amplicons at a ratio of 1:1 and 2:1, respectively (Figure 10A,B).

Thus, it seems necessary to get at least ~30% of edited transcript in the cell to visualize a significant G peak in the electropherogram by standard DNA sequencing.

## 3. Discussion

We used the REPAIRv2 tool to edit a premature stop codon (UGA) in different cell models with the aim of recovering full-length proteins. Collectively, our results suggest, for the first time to our knowledge, that the REPAIRv2 tool is able to edit the UGA premature stop codon present in the HeLa-H2BGFP*^opal^* and FRT-CFTR*^W1282X^* engineered cells, as well as the UGA premature stop codon present in IB3-1CF human airway epithelial cells (CFTR F508del/W1282X).

However, following editing assays, we detected a moderate-intensity signal of direct green fluorescence in a small number of HeLa-H2BGFP*^opal^* cells and of immunofluorescence in FRT-CFTR*^W1282X^*. Moreover, standard DNA sequencing technique was unable to detect TGA>TGG conversion, as revealed by the absence of the expected double A/G peak at target position, in Hela-H2BGFP*^opal^* and FRT-CFTR*^W1282X^* cells. This result suggests that the total amount of edited transcript was likely under the threshold of sensitivity of the technique. In fact, we observed the expected double peak with a 2:1 ratio (WT:mutant amplicons), indicating that it is necessary to get at least 30% of edited transcript to visualize the G peak in the electropherogram by standard DNA sequencing. These results reflect both the transfection efficiency, which is usually around 20–30%, and the low level of nonsense transcript in engineered cells, which could also differ cell by cell. Indeed, in these cells, H2BGFP*^opal^* and *CFTR^W1282X^* nonsense mRNAs probably underwent the mRNA NGD pathway.

In IB3-1 cells, the transcript encoded from the *CFTR* allele with the W1282X nonsense mutation was degraded by the nonsense-mediated decay (NMD) pathway [31], protecting cells from translation of the mutant transcript, but here *CFTR^W1282X^* mRNA was endogenously expressed. Indeed, we could see a signal indicating rescued CFTR in more cells than in FRT cells. Nevertheless, it was still not possible to quantify the CFTR protein by Western blot (data not shown).

Considering the complexity of the REPAIRv2 technique, we think that the number of cells that undergo the mRNA editing, which are detected by IF or direct fluorescence, was likely not enough to obtain a CFTR or H2BGFP protein amount to be detected by Western blot in total extracts. A full-length H2BGFP protein from H2BGFP*^opal^* HeLa cells was detected by Western blot, but only after immunoprecipitation with anti-GFP antibody (Figure S8A,B). Anyway, the immunofluorescence results highlighted by the IF images’ quantification of FRT-CFTR*^W1282X^* and IB3-1 cells (Figure 7B and Figure 8B) suggest that editing of the UGA premature termination codon in these cells resulted in the partial restoration of the CFTR protein. In addition, it seems that rescued CFTR properly localized onto the plasma membrane. However, it remains to be determined if the protein’s channel function was recovered.

From our results, we could not reason about the eventual off-target activity, which will need to be rigorously investigated in the future. However, one of the most attractive features of REPAIRv2 is the use of mutant hADAR2_DD_ (E488Q/T375G), which showed the highest level of editing associated with the lowest numbers of transcriptome-wide off targets when compared to other RNA editing approaches.

Finally, though transcript editing seems to be modest, by looking at the immunofluorescence results, we believe that the REPAIRv2 system is potentially suitable for the correction of UGA PTC present in *CFTR^W1282X^* transcript and we hypothesize that the REPAIRv2 editing tool could get advantage by reagents that increase/stabilize the transcript to be edited.

## 4. Materials and Methods

### 4.1. Site-Directed Mutagenesis and Bacterial Clone Selection

Site-directed mutagenesis was performed with the Quick change II site-directed-mutagenesis kit (Agilent) based on a few rounds of PCR amplification of the plasmid harboring target DNA with a high-fidelity DNA polymerase and the following couple of complementary mutant primers: W1282Xop-dw and W1282Xop-up. Unmethylated, newly synthesized plasmids were transformed into super-competent XL1Blue bacterial strain. Colonies harboring the pTracer-CFTR^W1282X^ were identified by colony PCR with primers CFTRdw3 and CFTRup4. Plasmid DNA was purified from a positive colony and sequenced with primer CFTRup4. Nucleotide substitution g3846a was confirmed by DNA sequencing (BMR genomics). Oligonucleotides used in this subsection are listed in Table S1.

### 4.2. Vectors and Clones Used in This Study

The sequence coding for the fusion protein was expressed from the CMV-dPspCas13b-GS-ADAR2_DD_ (E488Q/T375G)-delta-984-1090 vector (Addgene). The specific *g*RNA was transcribed under the control of the U6 promoter after cloning the DNA fragment coding for the *spacer* into the PspCas13b crRNA backbone vector (Addgene), upstream of the hairpin coding sequence (Figure S3).

### 4.3. Spacer Cloning

The complementary oligonucleotides to be annealed, and subsequently cloned in PspCas13b crRNA backbone vector, were heated up to 94 °C and then allowed to cool down to room temperature in a medium salt buffer. After digestion with BbsI restriction enzyme and agarose gel purification, the PspCas13b crRNA backbone vector was ligated by T4 ligase with each of the DNA fragments separately and then transformed into XL_1_ Blue bacterial competent strain. Positive clones were selected by colony PCR with the primer pUC-M13 Rev coupled with GFP 3′G wt for GFP *g*RNA clones or −40 forw for CFTR *g*RNA clones. Plasmid DNA of clones selected for further characterization was extracted by a commercial kit (Invitrogen or Qiagen), gel verified, and sequenced with M13 Rev primer (BMR genomics). Oligonucleotides used to generate *g*RNA coding fragments are listed in Table S2. Oligonucleotides used for colony PCR and sequencing are listed in Table S3.

### 4.4. Cell Culture Conditions

The IB3-1 is a bronchial cell line derived from a cystic fibrosis patient with a F508del/W1282X CFTR genotype. IB3-1 and HeLa cells were cultured in Dulbecco's Modified Eagle Medium (DMEM) supplemented with 10% fetal bovine serum (FBS), 100 U/mL penicillin, 100 μg/mL streptomycin, and 1 mM sodium pyruvate. Fisher Rat Thyroid (FRT) cells were cultured in Coon’s modified F12 medium supplemented with 10% FBS, 2 mM glutamine, and 2.68 g/L sodium bicarbonate. Cells were grown in a humidified atmosphere of 5% CO_2_ in air at 37 °C.

### 4.5. Cell Transfection

HeLa-H2BGFP, FRT-CFTR, and IB3-1 cells grown on ibidi™ flasks were co-transfected at 90% confluency by Lipofectamine 3000 (Invitrogen) with plasmids coding for the different *guide* RNAs and the plasmid coding for dCAS13b/ADAR2_DD_. The ratio DNA/number of cells was calculated in accordance with the Lipofectamine 3000 protocol (Invitrogen). We transfected 300 or 450 ng of total DNA in HeLa cells and 150 or 300 ng of total DNA in FRT and IB3-1 cells, always maintaining the ratio 2:1 of gRNA:CAS13b/ADAR2DD, also when guides where used in combination.

### 4.6. Immunofluorescence

Forty-eight h post transfection, cells were fixed with methanol for 5′ and, when specified, they were further permeabilized with Triton 0.01% for 10′ a room temperature. Cells were then blocked with bovine serum albumin (BSA) 0.1% at R.T. for 30′ and incubated with the mAb570 (provided by the University of North Caroline) or the mAbCF3 (mAb 2784 Abcam) and diluted 1:500 in BSA 0.1% overnight at 4 °C. They were finally incubated with secondary anti-mouse mAb (FITC-conjugated, Sigma, diluted 1:300 or Alexa 488, Abcam, diluted 1:500) for 1 h at room temperature before DAPI staining and microscopy observation. The exposure time was adjusted using the negative control as reference (no fluorescence signal observed).

### 4.7. RT-qPCR

Total RNA was extracted by using the RNAeasy Mini Kit (Qiagen) and retrotranscribed with High-Capacity cDNA Reverse Transcription Kit (Applied Biosystem). The cDNAs (50 ng/replicate) were then added to a mix containing SYBR^TM^ Green (Applied Biosystems; 12,5 µL/replicate), H_2_O, for a final volume of 20 µL/well and forward and reverse primers 1 µM each (CFTRdw10 5′acttctaatggtgatgacagcc3′/CFTRup9 5′-atccagcaaccgccaacaact3′; GFP-F 5’agaacggcatcaaggtgaac3’/ GFP-R 5’tgctcaggtagtggttgtcg3’). Samples were analyzed with the thermocycler Applied Biosystems 7300 Real Time PCR System 96-well qPCR (15″ at 95 °C, 60″ at 60 °C, for 40 cycles) [36,37].

### 4.8. The cDNA Synthesis and RT-PCR

The cDNAs were amplified by standard PCR with specific primers annealing to sequences close to the editing target site. The cDNAs from HeLa- and FRT-transfected cells were amplified with GFPdown/GFPrev and CFTRdw3/CFTRup4 couples of primers, respectively. Sequencing was performed by BMR genomics with GFPrev or CFTRdw3 primers. Primers are listed in Tables S1 and S3.

### 4.9. Western Blotting and Immunoprecipitation

Two days after transfection, HeLa-H2BGFP*^opal^* cells were lysated with RIPA Lysis and Extraction buffer (ThermoFisher Scientific, MA, USA) as previously described [38,39,40]. Then, 20 µg of total proteins were separated on a 10% polyacrylamide (SDS-PAGE) and transferred onto a nitrocellulose membrane. Membrane was then blocked with 5% milk in tween tris-buffered saline (T-TBS) solution for 1 h at room temperature and incubated overnight at 4 °C with a mouse monoclonal antibody anti GFP (1:1000) (Santa Cruz, CA, USA). Following incubation with an anti-mouse peroxidase-linked antibody (1:5000), the reaction was revealed by the enhanced chemiluminescence (ECL) detection system, using chemidoc imaging system (Biorad). For the immunoprecipitation, the total cellular lysates from two independent experiments were pooled and dialyzed against H_2_O for 48 h at 4 °C. Dynabeads^®^ (3 mg) were incubated with the mouse monoclonal antibody anti-GFP (10 μg) in phosphate buffered saline (PBS) (pH 7.4) with 0.01% Tween^®^-20 for 1 h under rotation. The proteins were incubated with rotation for 1 h at room temperature with the Dynabeads^®^-Ab complex. Surnatants (unbound) and proteins eluted off the beads (bound) were separated on an 8% polyacrylamide gels (SDS-PAGE), transferred onto a nitrocellulose membrane, and analyzed by Western blotting as above.

### 4.10. Quantifications of the Immunofluorescence

The quantifications of immunofluorescence experiments were relative to the replicate representative of the results. Quantification of CFTR signal was done manually by using Fiji. Briefly, the cell contour was drawn by using a cell membrane marker. The background was subtracted and the integrated signal intensity or the pixel mean in the selected area (one cell) was measured.

## Supplementary Materials

Supplementary materials can be found at https://www.mdpi.com/1422-0067/21/13/4781/s1. Figure S1: Spacer sequences for H2BGFP*^opal^* (W190X) editing. Figure S2: Spacer sequences for *CFTR^W1282X^* editing. Figure S3: Vectors for CRISPR-based RNA editing. Figure S4: Colony PCR to select positive GFP and CFTR *g*RNA clones. Figure S5: Site-directed mutagenesis. Figure S6*:* Immunofluorescence analysis of CFTR protein in sole methanol fixed FRT-CFTR^W1282X^ cells and quantification of the immunofluorescence. Figure S7: Immunofluorescence analysis to detect the CFTR protein in in sole methanol fixed IB3-1 cells and quantification of the immunofluorescence. Figure S8: Western blotting analysis to detect H2BGFP protein after editing of the PTC (UGA) in H2BGFP*^opal^* mRNA expressed in HeLa cells. Table S1: Oligonucleotides used for site-directed mutagenesis, colony PCR, and sequencing described in Section 4.1. Table S2: Oligonucleotides used to generate *g*RNA coding fragments. Table S3: Oligonucleotides used for colony PCR and sequencing described in Section 4.3 and RT-PCR in Section 4.7.
