# Supplementary material for: Investigating REPAIRv2 as a Tool to Edit CFTR mRNA with Premature Stop Codons
Source: Int J Mol Sci. 2020 Jul 6;21(13):4781. doi: 10.3390/ijms21134781 (PMC7369808; doi:10.3390/ijms21134781)
Supplement: Supplementary file 1 [file ijms-21-04781-s001.pdf]

## Supplementary Materials

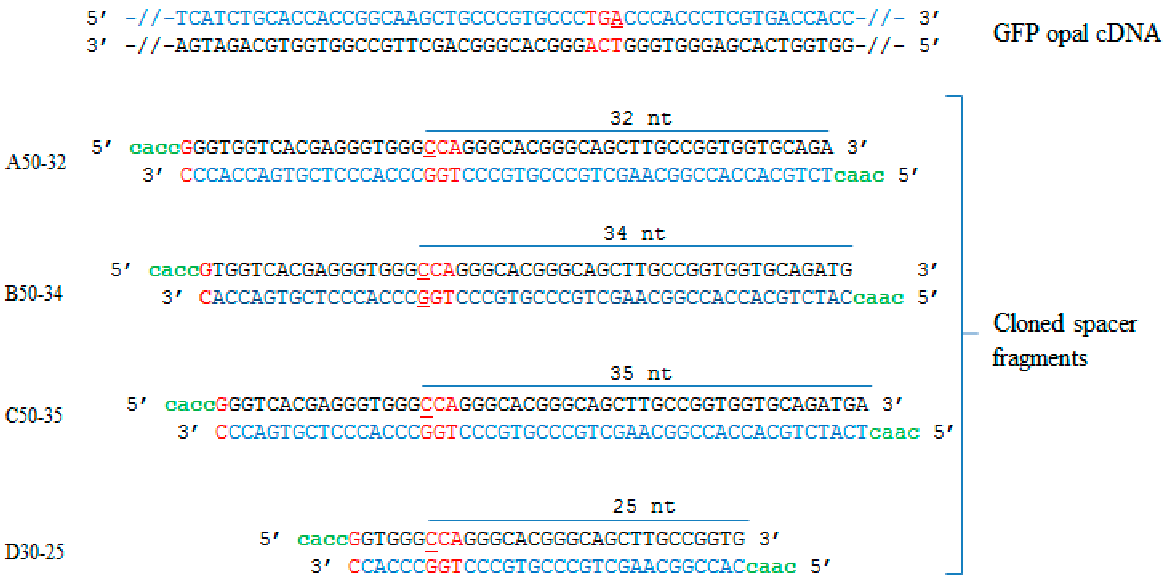


**Figure S1.** Spacer sequences for H2BGFP*opal* (W190X) editing. The nucleotide distance of mismatched C (underlined) from the 3′ end is shown. Blue: 5′-3′ GFP sense sequence. Red: the 5′ G and target codon. Target adenine is underlined. Green: BbsI restriction site overhangs. Top: reference sequence of mutant H2BGFP*opal* cDNA.

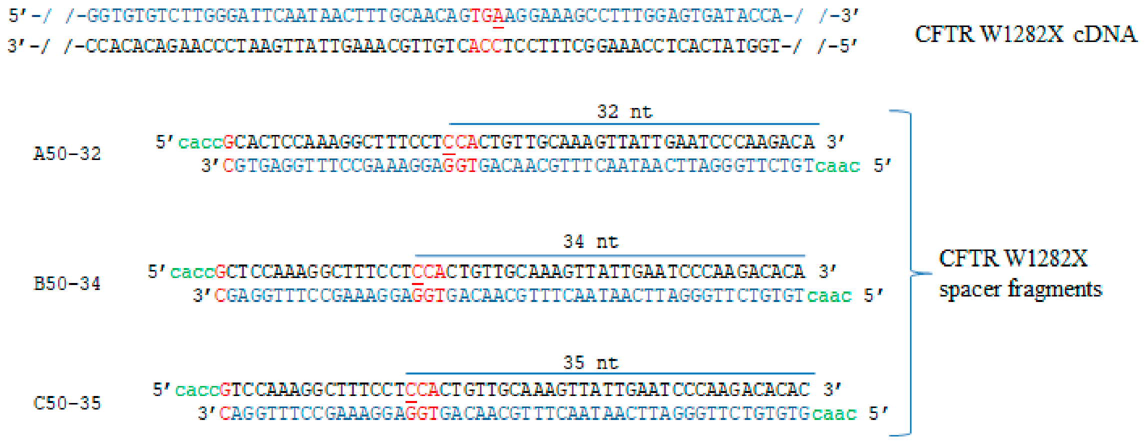


**Figure S2**. Spacer sequences for CFTR*W1282X* editing. The nucleotide distance of mismatched C (underlined) from the 3′ end is shown. Blue: 5′-3′ CFTR sense sequence. Red: the 5′ G and target codon. Target adenine is underlined. Green: BbsI restriction site overhangs. Top: reference sequence of CFTR*W1282X* cDNA.

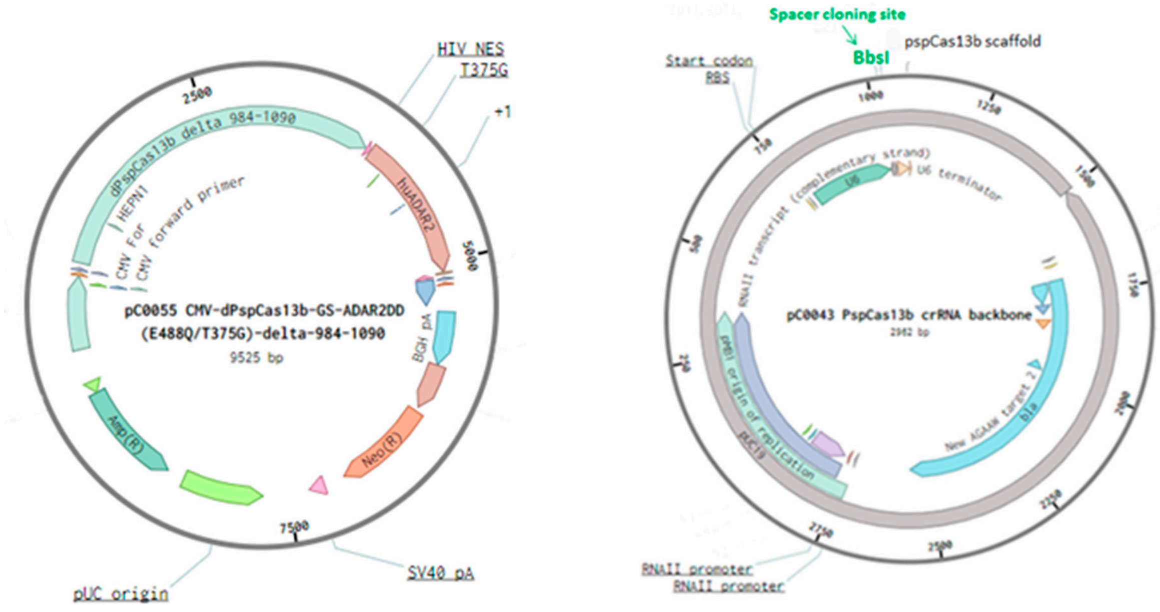


**(A)** **(B)**

**Figure S3.** Vectors for CRISPR based RNA editing. (**A**) CMV-dPspCas13b-GS-ADAR2DD (E488Q/T375G)-delta-984-1090 vector. (**B**) PspCas13b crRNA backbone vector; green arrow points the spacer cloning site BbsI. Modified from Addgene.

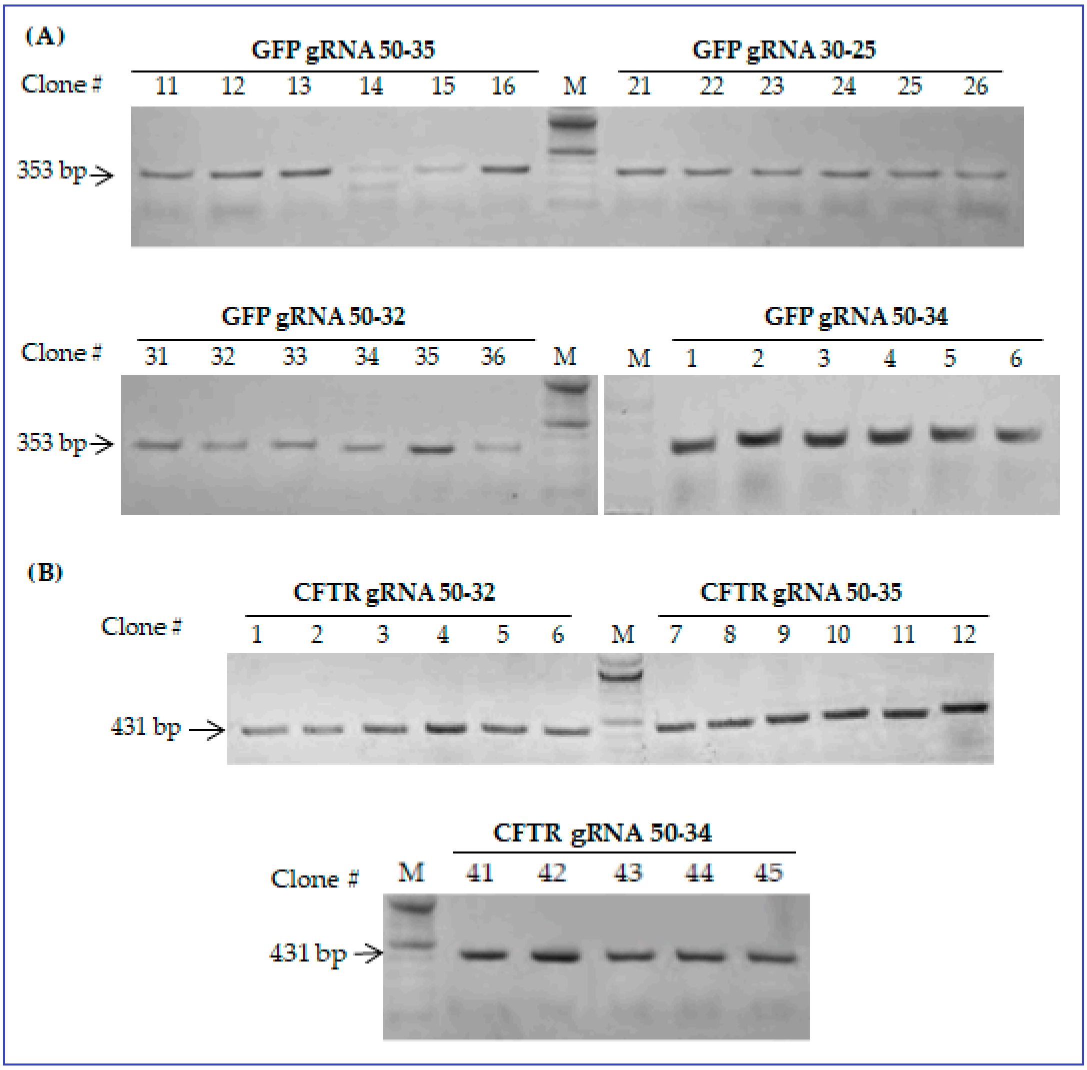


**Figure S4**. Colony PCR to select positive GFP and CFTR *g*RNA clones. For GFP we used primers M13rev/GFP 3′ Gwt (**A**). For CFTR *g*RNA clones with primers M13Rev/-40 forw. (**B**) The following clones were purified, sequenced and used for further investigations: GFP *g*RNA 50-34 #1, GFP *g*RNA 50-35 #11, GFP *g*RNA 30-25 #21 and GFP *g*RNA 50-32 #31, CFTR *g*RNA 50-34 # 41, CFTR *g*RNA 50-32 #4 and CFTR *g*RNA 50-35 #7. Clone name and number and amplicon size are shown. M: 2log ladder (Biolabs).

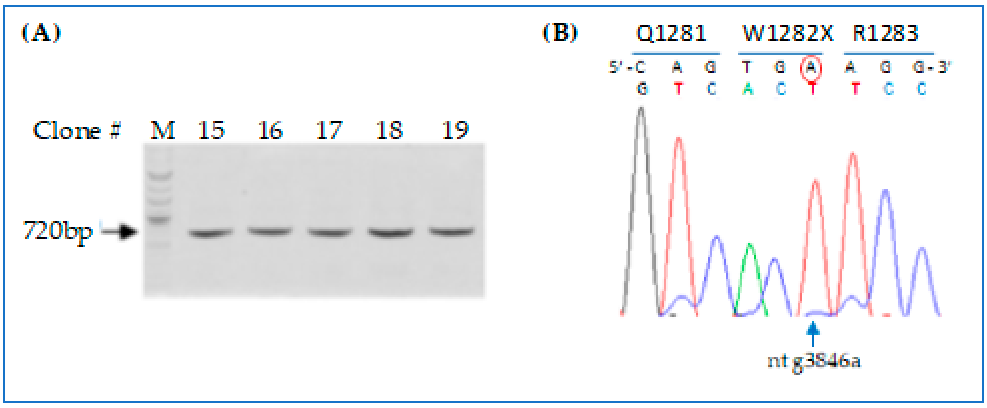


**Figure S5.** Site directed mutagenesis. (**A)** Colony PCR with primers CFTR 3dw/CFTR up4 to select colonies with the vector and (**B**) Electropherogram showing the CFTR$^{W1282X}$ mutation in clone #16.

**(A)**

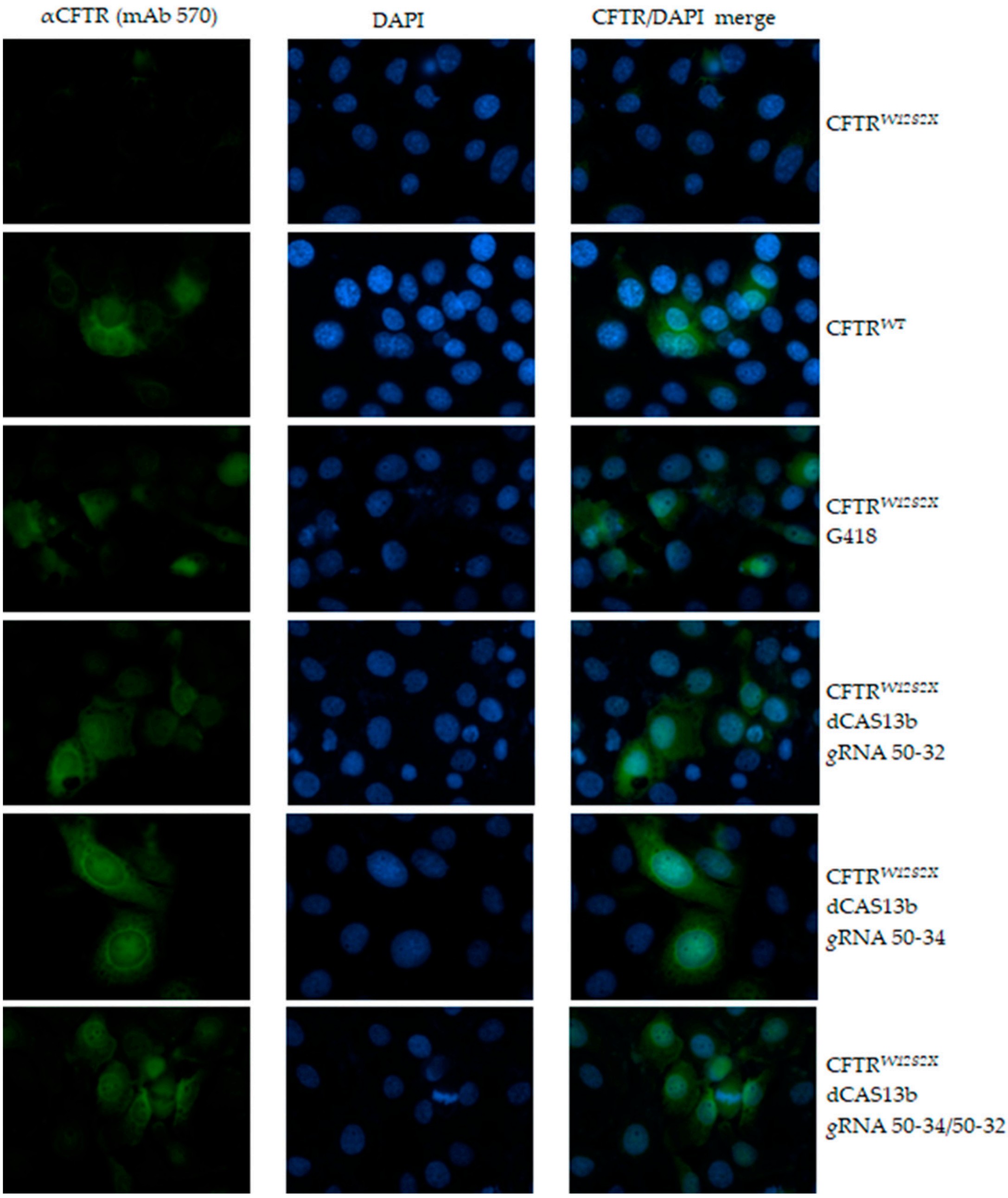
αCFTR (mAb 570)
DAPI
CFTR/DAPI merge
$CFTR^{W1282X}$
$CFTR^{WT}$
$CFTR^{W1282X}$
G418
$CFTR^{W1282X}$
dCAS13b
gRNA 50-32
$CFTR^{W1282X}$
dCAS13b
gRNA 50-34
$CFTR^{W1282X}$
dCAS13b
gRNA 50-34/50-32

**(B)**

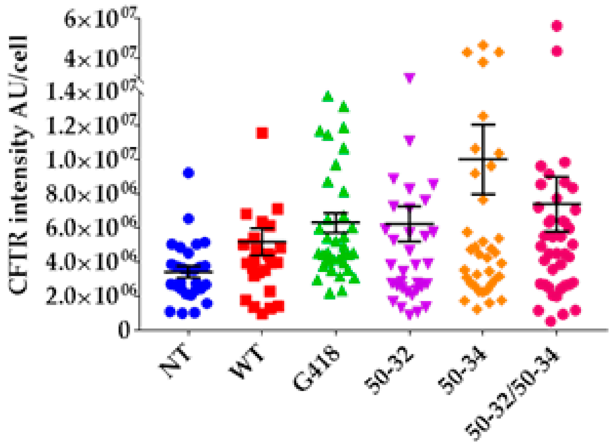


**Figure S6.** Immunofluorescence analysis of CFTR protein in sole methanol fixed FRT-CFTR$^{W1282X}$ cells and quantification of the immunofluorescence. FRT-CFTR$^{W1282X}$ cells were transfected with the plasmids encoding the indicated *g*RNAs and dCAS13b/ADAR2$_{DD}$ (300 ng total DNA). Untransfected FRT-CFTR$^{W1282X}$ cells (NT) were used as negative control. FRT-CFTR$^{W1282X}$ cells treated with G418 and FRT-CFTR$^{WT}$ cells were used as a positive control. (**A**) The CFTR protein was revealed by the primary antibody mAb570 followed by a secondary antibody anti-mouse-FITC conjugated (green, Sigma). Nuclei (blue) were DAPI stained. Images were taken at 63x magnification on a ZEISS microscope equipped for epifluorescence. (**B**) The quantification is relative to the replicate representative of the results. The error bar represents the SEM. The quantification of CFTR signal was done manually by using Fiji software. The cell contour was drawn by using a cell membrane marker. The background was subtracted and the integrated signal intensity in the selected area (one cell) was measured.

(A)

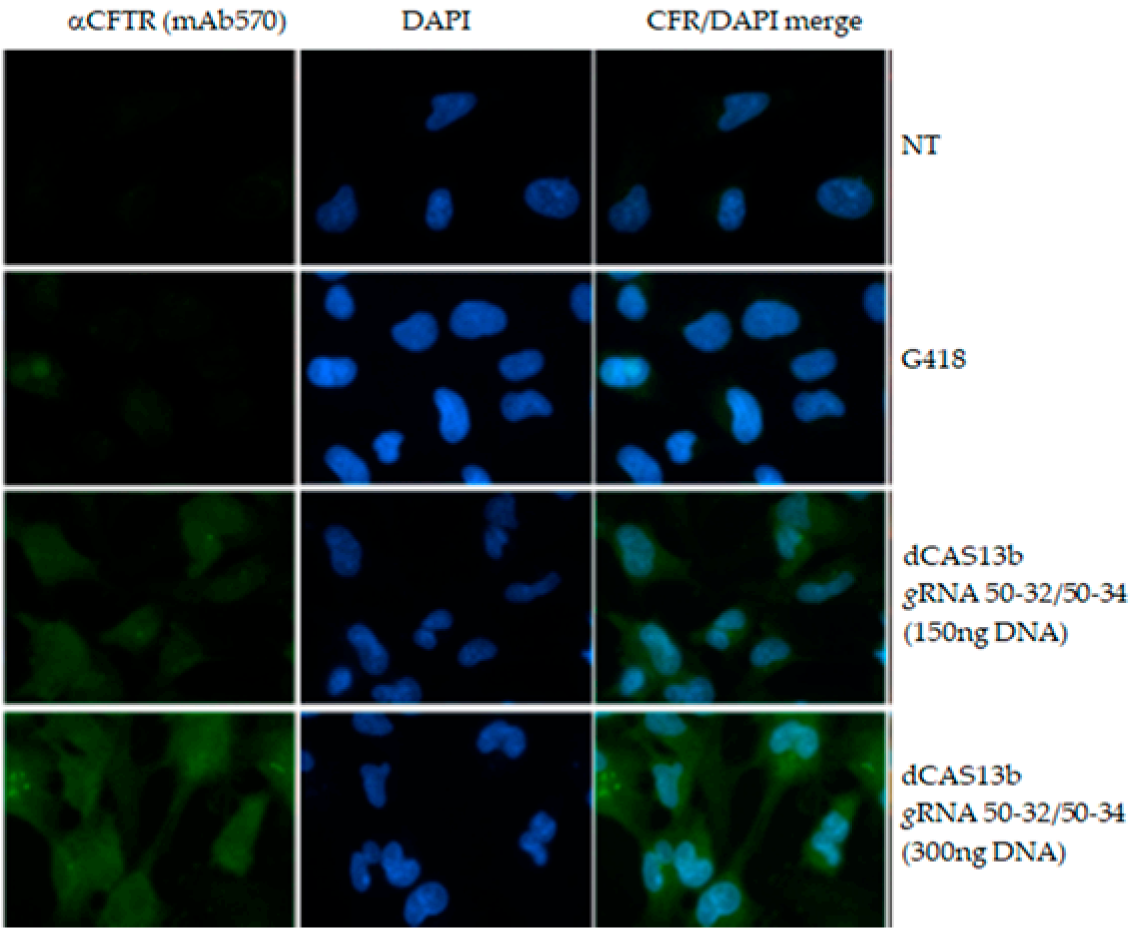


(B)

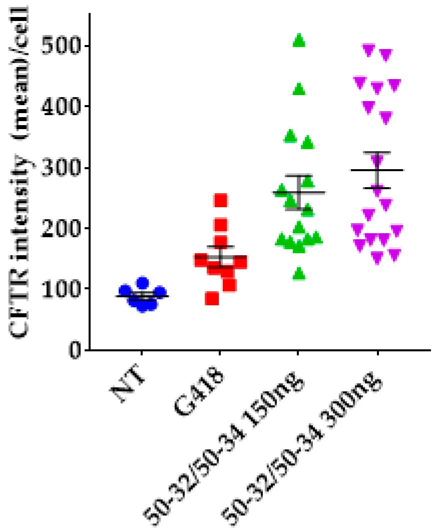


**Figure S7.** Immunofluorescence analysis to detect the CFTR protein in sole methanol fixed IB3-1 cells and quantification of the immunofluorescence. IB3-1 cells were untreated (NT: negative control), treated with G418 (positive control) or transfected with the plasmids encoding the 50-32/5034 *g*RNAs and dCAS13b/ADAR2$_{DD}$. **(A)** CFTR protein was revealed by the specific primary antibody mAb570 and a

secondary antibody (green, Alexa-488, Abcam). Nuclei (blue) were DAPI stained. Images were taken at 63x magnification on a ZEISS microscope equipped for epifluorescence. **(B).** The quantification is relative to the replicate representative of the results. The error bar represents the SEM. The quantification of CFTR signal was done manually by using Fiji software. The cell contour was drawn by using a cell membrane marker. The background was subtracted and the pixel mean in the selected area (one cell) was measured.

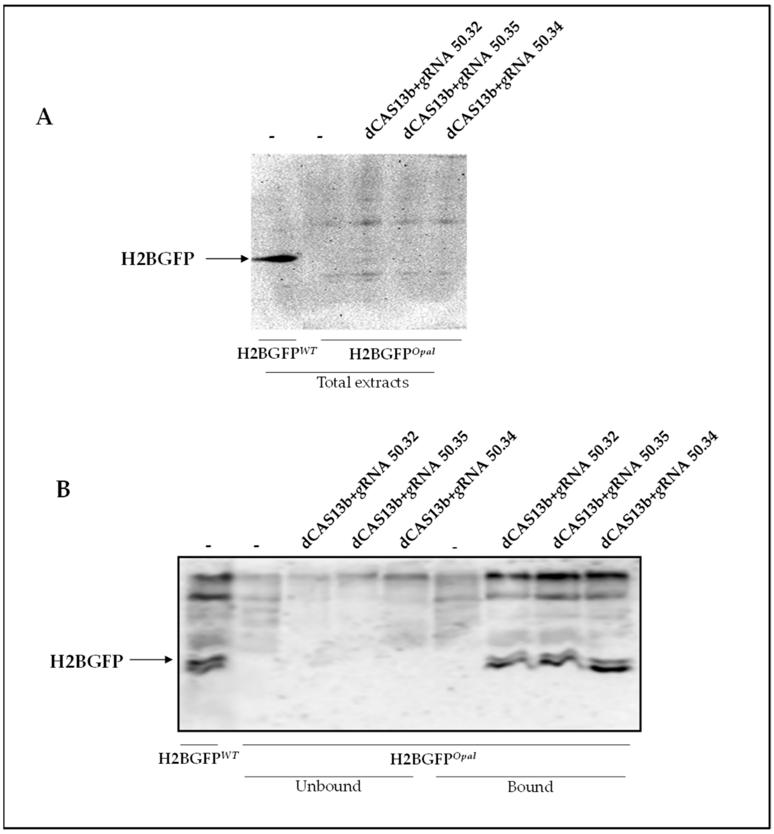


**Figure S8**. Western Blotting analysis to detect H2BGFP protein after editing of the PTC (UGA) in H2BGFP$^{opal}$ mRNA expressed in HeLa cells**.** (**A**) cells were co-transfected with dCAS13b/ADAR2$_{DD}$ and indicated *g*RNAs coding plasmids (450 ng total DNA), HeLa-H2BGFP$^{opal}$. Two days after transfection the cells were lysated with RIPA buffer, as previously described [35-37]. 20μg of total proteins were separated on a 10% polyacrylamide (SDS-PAGE) gel under reducing conditions and transferred onto a nitrocellulose membrane. Membrane was then blocked with 5% milk in T-TBS solution for 1 h at room temperature and incubated overnight at 4°C with a mouse monoclonal antibody for GFP (1:1000) by Santa Cruz Biotechnology (Santa Cruz, CA, USA). Following incubation with an anti-mouse peroxidase-linked antibody (1:5000), the reaction was revealed by the ECL detection system, using chemidoc imaging system (Biorad). (**B**) The total cellular lysates from two independent experiments were pooled and dialyzed against $H_2O$ for 48h at 4°C. Dynabeads® (3 mg) were incubated with the mouse monoclonal antibody anti GFP (10 μg) in PBS (pH 7.4) with 0.01% Tween®-20 for 1h under rotation. The proteins were incubated with rotation for 1h at room temperature with the Dynabeads®-Ab complex. Surnatants (Unbound) and proteins eluted off the beads (Bound), were separated on a 8% polyacrylamide gels (SDS-PAGE), transferred onto a nitrocellulose membrane and analyzed by Western blotting as above. HeLa H2BGFP$^{WT}$ cells and untransfected H2BGFP$^{opal}$ cells were used as positive and negative control, respectively.

| | |
|---|---|
| W1282Xop-dw | 5'-caataactttgcaacagtg**a**aggaaagcctttggagtga-3' |
| W1282Xop-up | 5'-tcactccaaaggctttcct**t**cactgttgcaaagttattg-3' |
| CFTRdw3 | 5'-tcatcttcttcattgctgttac-3' |
| CFTRup4 | 5'-ttaggacacgcccccatc-3' |

**Table S1.** Oligonucleotides used for site directed mutagenesis, colony PCR and sequencing described in *section 4.1*. dw: forward primer; up: reverse primer. In the sequences mutant nucleotides are in bold.

| | |
|---|---|
| GopC13 50-32 F | 5′ caccgggtggtcacgagggtgggccagggcacgggcagcttgccggtggtgcaga 3′ |
| GopC13 50-32 R | 5′ caactctgcaccaccggcaagctgcccgtgccctggcccaccctcgtgaccaccc 3′ |
| GopC13 50-35 F | 5′caccgggtcacgagggtgggccagggcacgggcagcttgccggtggtgcagatga 3′ |
| GopC13 50-35 R | 5′caactcatctgcaccaccggcaagctgcccgtgccctggcccaccctcgtgaccc 3′ |
| GpoC13 50-34 F | 5′ caccgtggtcacgagggtgggccagggcacgggcagcttgccggtggtgcagatg 3′ |
| GpoC13 50-34 R | 5′ caaccatctgcaccaccggcaagctgcccgtgccctggcccaccctcgtgaccac 3′ |
| GopC13 30-25 F | 5′caccggtgggccagggcacgggcagcttgccggtg 3′ |
| GopC13 30-25 R | 5′ caaccaccggcaagctgcccgtgccctggcccacc 3′ |
| CFw1282x 50-32 F | 5′caccgcactccaaaggctttcctccactgttgcaaagttattgaatcccaagaca 3′ |
| CFw1282x 50-32 R | 5′caactgtcttgggattcaataactttgcaacagtggaggaaagcctttggagtgc 3′ |
| CFw1282x 50-35 F | 5′ caccgtccaaaggctttcctccactgttgcaaagttattgaatcccaagacacac 3′ |
| CFw1282x 50-35 R | 5′ caacgtgtgtcttgggattcaataactttgcaacagtggaggaaagcctttggac 3′ |
| CFw1282x 50-34 F | 5′ caccgctccaaaggctttcctccactgttgcaaagttattgaatcccaagacaca 3′ |
| CFw1282x 50-34 R | 5′ caactgtgtcttgggattcaataactttgcaacagtggaggaaagcctttggagc 3′ |

**Table S2.** Oligonucleotides used to generate *g*RNA coding fragments. GopC13 were used for GFP gRNA, CFw1282x for CFTR *g*RNA. F and R: forward and reverse primer respectively. Numbers in the name of oligonucleotides refer to the distance from the 3′ end of the spacer of the C missmatch (in red) in front of the target adenine.

| | |
|---|---|
| pUC-M13 Rev | 5′-agcggataacaatttcacacagg-3′ |
| GFP 3′G wt | 5′-agctgcccgtgccctgg-3′ |
| -40 forw | 5′-gttttcccagtcacgacgttg-3′ |
| GFPdown | 5′cgtaaacggccacaagtt3′ |
| GFPrev | 5′cgacaaccactacctgagca3 |

**Table S3.** Oligonucleotides used for colony PCR and sequencing described in *subsection 4.3* and RT-PCR *in section 4.7.*
